# Mr. Ward, in Reply to Mr. G. N. Hill

**Published:** 1804-02-01

**Authors:** M. Ward

**Affiliations:** Manchester


### *To the Editors of the Medical and Physical Journal*

GENTLEMEN,

WHEN I entered on the consideration of the medicinal properties of opium, I did not expect, neither did I wish, a question so complicated and interesting to be decided without an ample discussion of its merits; but I did wish to avoid useless and unnecessary altercation about extraneous matters; you will therefore, I trust, give me credit for the assertion when I say, it is with no small concern that I have occasion to call your attention and that of your readers, to two communications inserted in the 48th and 58th Numbers of the M. and P. Journal, signed, George Nesse Hill.

The *desultory* manner in which the subject is there treated would have justified me in allowing them to pass unnoticed, had not the author accused me of inculcating a doctrine, trine, which I have uniformly and strenuously opposed; and when called upon to adduce proofs in support of his assertions, (which it was certainly incumbent upon him, as a candid man, to have done,) instead of complying with a request so reasonable, and one, which, could it have been done at all, might have been done with very little trouble; allows nine months to elapse without taking any notice of it; and when at the expiration of that time, his next paper appears, in which I expected to have seen the charge proved or retracted, I find no mention is made either of his former assertions or of my request; but, on the contrary, that it commences with an *erroneous statement of a passage from one of my papers.*

There is such an evident want of candour in this mode of proceeding, as, in my opinion, to disqualify Mr. Hill (even supposing him to be in other respects competent) for the performance of the task he has undertaken.

But I must now proceed to do that *justice* to myself which Mr. H. has so long and ungraciously withheld.

His accusation is conveyed in the following terms:

"It does not appear to me," (says Mr. Hill, Vol. x, page 154) "that this gentleman, when he first announced this matter to the public, conceived of the action of opium in the manner he has lately avowed, when employed in his critique on Dr. Crumpe's Inquiry; *for in some of his first communications he speaks of this drug as a tonic stimulant, in the latter ones as a sedative.*"

And in page 155, "We are not favoured with Mr. W's. definition of these important words, (stimulant and sedative) until we arrive at page 127 of Number 36; *and here I may be permitted to observe, that the accusation brought against the advocates for the stimulant doctrine, as Mr. W. calls it, is not less ascribable to those of an opposite opinion, this gentleman sometimes calling opium a tonic, at others a sedative.*"

And again, the third time, in page 157:

"*But, as already observed, Mr. W. has applied both these terms to opium; it is evident one or other of them must be abandoned, unless he can by any means prove, that the medicine possesses opposite qualities, or has diametrically opposite effects, according to the mode of its exhibition.*"

Now, I conceive it to be exceedingly improper for a writer to accuse another of inconsistency, without, *at the same time,* bringing proof in support of the accusation; but to withhold the proof when called upon to furnish it, is an offence against all the rules of decorum, and demands the most ample apology.

A charge

A charge of this nature certainly surprized me, *having taken some pains to shew that there is no foundation for the opinion implied in it*; and, were it necessary, I could easily bring many passages in support of what is here advanced, but shall content myself with the following from Vol. 7. p. 346.

"Dr. Crumpe's view in making the experiment seems evidently to have been, to observe the *local appearances* resulting from the application of a solution of opium to a tender irritable surface; and this accounts for his omitting to notice any other circumstance. But after having proved to a demonstration that the *primary and general* operation of opium injected into the cavity of the abdomen is directly and powerfully sedative (both the voluntary and involuntary motions yielding to its influence) it necessarily follows, that the increased redness and apparent inflammation, *could not have been the consequence of the opium having acted as a stimulant, unless we suppose that it acts as a stimulant and a sedative at the same time, which would be absurd; and indeed Dr. Crumpe has shewn that idea to be unfounded*." See his Inquiry, p. 167—8.

This however is only negative proof, but what I have written is upon record; and if there be any truth in Mr. Hill's charge, there can be no difficulty whatever in proving it.

Well knowing it to be unfounded, I added the following postscript to a letter I happened to be at that time preparing, and which appeared in the M. and P. Journal for April 1803. (Vol. ix. p. 348.)

"The request with which the above letter commences, precludes me from replying to Mr. Hill's communication, inserted in No. 48; I shall therefore only observe, that my sole view in selecting the quotation to which Mr. H. alludes, was merely to sanction the publication of the facts which had occurred to me, and that my choice was not directed by any opinion I had formed of the modus operandi of opium, (for I confess I had not then paid sufficient attention to the subject to enable me to make up my mind upon it) much less of its manner of operating in the disease treated of by Mr. Pott."

It will not, I hope, be deemed inconsistent with the tenor of my request, *to desire Mr. Hill will have the goodness to point out, through the medium of the Medical Journal, in which of my papers I have spoken of opium as a tonic stimulant, or have attributed opposite qualities to it.*

And expected to have found in the next, and every succeeding number, that it had been attended to; *Mr. Hill's reputation as a writer certainly requiring that an immediate and full answer should have been given.* In this expectation however I have been disappointed, Mr. H. having published another paper in the Journal for the present month, without adverting to it. *He has certainly quoted a passage from one of my papers; but by adding a word, and leaving out two, has entirely perverted its original meaning: and this misrepresentation* (as the paper commences with it) *seems to have been intended as a justification of his conduct.*

The passage as it stands in my paper (M. and P. J. Vol. vi. p. 480) is as follows:

"*After the same medicine* (opium) *both alone and joined with other antispasmodics, tonics, &c.*"

As represented, *or rather misrepresented* by Mr. Hill, it stands thus.

"*Opium joined with other antispasmodics, and tonics.*" See Vol. x. p. 532.

The most favourable construction that can be put upon Mr. Hill's conduct in this instance, is; that he quoted at random, without any regard to accuracy.

"*Can the best interests of the profession in any degree be furthered*" (to use a phrase of Mr. Hill's) "*by such unworthy means?* or, can such a proceeding, (to use another of Mr. Hill's) "*tend to the promotion of that desirable end of all amicable discussion,*" (truth and consistency)? *

It

---

* "Having taken some pains (says Mr. H. Vol. ix. p. 153—4) to understand the new theory of the modus operandi of opium, as attempted to be established by Mr. Ward of Manchester, and finding that every number of your useful work, containing any of that gentleman's remarks on this great subject, has tended only to convince me of the just foundation in truth of Dr. Crumpe's statement, *although that author's work has not* FALLEN *into my hands)* I have taken the liberty of troubling you with a few occasional thoughts, which have occurred to me on this important matter; *if you think the best interests of our profession will in any degree be furthered by the insertion of them, I crave a place in the Journal, at your convenience; if not, please to set them aside.*"

"I have, for some time, been waiting with some degree of impatience, to see the doctrine Mr. W. has endeavoured to elucidate, and enforce, freely canvassed and discussed in all its views, *that its harmony with truth, and its consistency with experience,* might establish the fact, or its fallacy and speciousness be detected, and the question be for ever set at rest. Hitherto I have been disappointed; *it will give me no trifling satisfaction to find that what I have said has tended to the promotion of this desirable end*

It may perhaps be said, that Mr. Hill's last paper has been written some time, and kept back in consequence of my desiring the readers of the Journal to suspend their remarks on my papers, until the whole of the evidence shall have been laid before them; but this plea will be of no avail, as it contains nothing to the purpose.

It only remains for me to apologize to yourselves and your readers, for having occupied so much of their and your attention on so unpleasant an occasion.

*Manchester,* I am, &c.

*December* 22, 1803. M. WARD.

*end of all amicable discussion,* being long since assured that the real interests of truth have nothing to apprehend from the closest severity of investigation, nor the utmost censure human judgment can pronounce."

